# Wetting Simulations of High-Performance Polymer Resins on Carbon Surfaces as a Function of Temperature Using Molecular Dynamics

**DOI:** 10.3390/polym13132162

**Published:** 2021-06-30

**Authors:** Swapnil S. Bamane, Prashik S. Gaikwad, Matthew S. Radue, S. Gowtham, Gregory M. Odegard

**Affiliations:** Department of Mechanical Engineering—Engineering Mechanics, Michigan Technological University, Houghton, MI 49931, USA; ssbamane@mtu.edu (S.S.B.); prashikg@mtu.edu (P.S.G.); msradue@mtu.edu (M.S.R.); g@mtu.edu (S.G.)

**Keywords:** surface tension, computational simulation, resin selection, processability

## Abstract

Resin/reinforcement wetting is a key parameter in the manufacturing of carbon nanotube (CNT)-based composite materials. Determining the contact angle between combinations of liquid resin and reinforcement surfaces is a common method for quantifying wettability. As experimental measurement of contact angle can be difficult when screening multiple high-performance resins with CNT materials such as CNT bundles or yarns, computational approaches are necessary to facilitate CNT composite material design. A molecular dynamics simulation method is developed to predict the contact angle of high-performance polymer resins on CNT surfaces dominated by aromatic carbon, aliphatic carbon, or a mixture thereof (amorphous carbon). Several resin systems are simulated and compared. The results indicate that the monomer chain length, chemical groups on the monomer, and simulation temperature have a significant impact on the predicted contact angle values on the CNT surface. Difunctional epoxy and cyanate ester resins show the overall highest levels of wettability, regardless of the aromatic/aliphatic nature of the CNT material surface. Tetrafunctional epoxy demonstrates excellent wettability on aliphatic-dominated surfaces at elevated temperatures. Bismaleimide and benzoxazine resins show intermediate levels of wetting, while typical molecular weights of polyether ether ketone demonstrate poor wetting on the CNT surfaces.

## 1. Introduction

Polymer matrix composite (PMC) materials are widely used as structural materials in aerospace and aeronautical vehicles. A large number of high-performance polymers have been developed for use as the matrix in PMCs with various carbon reinforcements such as carbon fiber, carbon nanotubes (CNTs), and graphene nano-sheets. Recently, CNTs have shown exceptional mechanical properties and are being considered as a reinforcement material for the next generation of ultralight high-strength composite materials [1,2]. Many decades of experimental-based research have addressed the compatibility of various polymer resins with the surface of carbon-based reinforcements [3,4]. Additionally, previous Molecular Dynamics (MD)-based computational studies have addressed the interaction of various polymer materials with carbon-based reinforcement materials [4,5,6,7,8,9,10]. However, a comprehensive understanding of such compatibility is lacking for the next generation of high-performance carbon-reinforced composite materials, which can simultaneously include reinforcement with aromatic and aliphatic carbon phases [11,12,13].

Wettability of resin onto the reinforcement surface is a key parameter in the manufacturing of PMCs. Determining the contact angle between specific combinations of liquid resin droplets and reinforcement surfaces is a common method to quantify wettability. However, the experimental measurement of contact angle can be difficult or cost prohibitive when screening multiple high-performance resins with CNT-based materials such as CNT bundles or yarns. Furthermore, the interpretation of experimental wetting results for some CNT materials is complicated by the presence of regions of amorphous carbon (mixture of aromatic and aliphatic carbon) on the aromatic CNT surface. Efficient computational prediction of contact angle could significantly facilitate the resin selection process for the design and development of new CNT-based PMCs.

Molecular Dynamics (MD) simulation has been previously used to study the wetting properties of water and polymer resin systems on surfaces such as graphite and metals [14,15,16,17,18,19,20,21,22]. These studies offer a good starting point for understanding how MD can be used to efficiently determine the contact angle and provide physical insight into the wetting process. However, a comprehensive study of wetting of different high-performance resins on CNT surfaces using MD simulation has not been performed. Therefore, a comprehensive MD-based study is needed to efficiently determine the relative wettability of different high-performance resins on CNT surfaces with the potential presence of regions of amorphous carbon. 

The objective of this study is to use MD simulation to predict the relative levels of wettability of various high-performance resins onto CNT surfaces. All-atom simulations are used to determine the contact angle with aromatic and aliphatic carbon surfaces for a range of temperatures to provide guidelines for resin selection and process design. In the following sections, the details of the materials and the modeling procedures are outlined. The results of the simulations are subsequently presented for a comprehensive understanding of resin wettability with CNT surfaces. The results indicate that resin and temperature selection can have a significant impact on resin wettability. 

## 2. Materials and Methods

### 2.1. Materials

**Figure 1:**
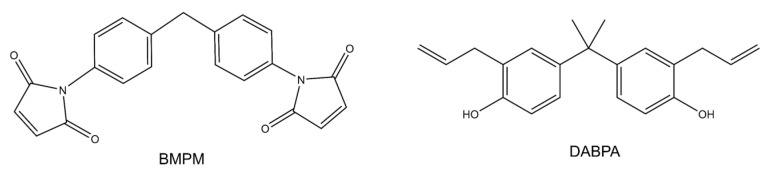
Molecular structure of BMI.

**Figure 2:**
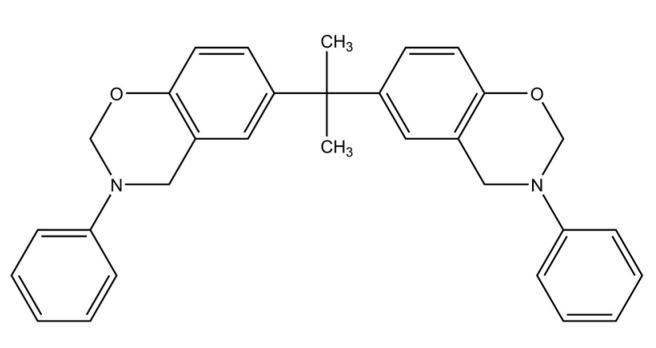
Molecular structure of benzoxazine.

**Figure 3:**
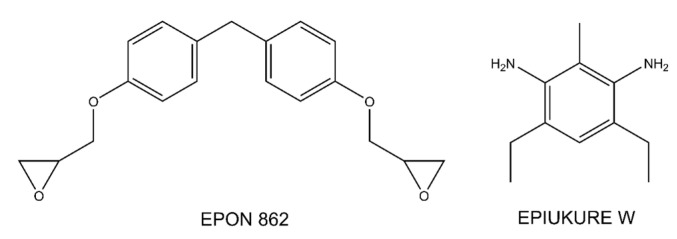
Molecular Structure of difunctional epoxy.

**Figure 4:**
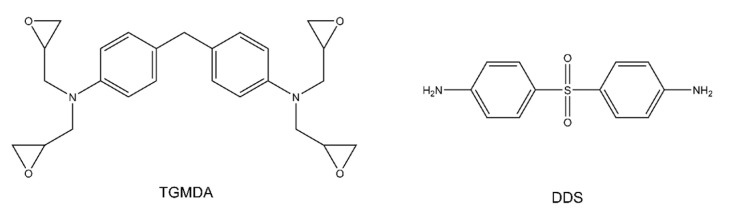
Molecular structure of tetrafunctional epoxy.

**Figure 5:**
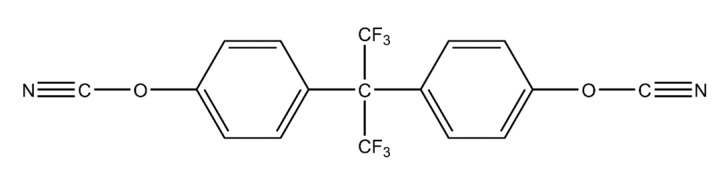
Molecular structure of fluorinated cyanate ester.

**Figure 6:**
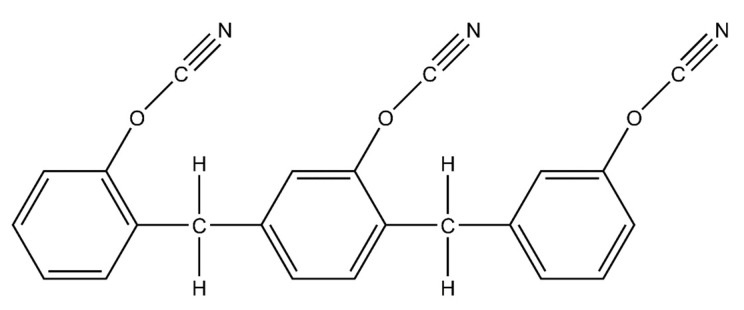
Molecular structure of non-fluorinated cyanate ester.

**Figure 7:**
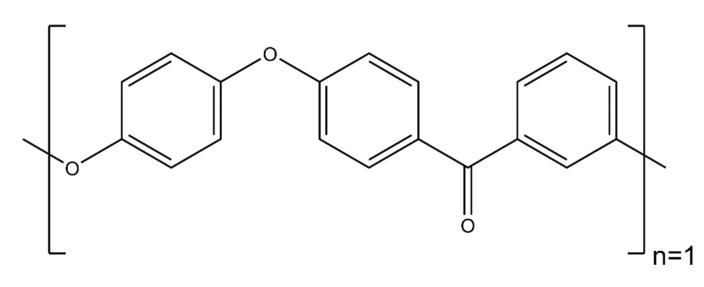
Molecular structure of peek monomer.

**Figure 8:**
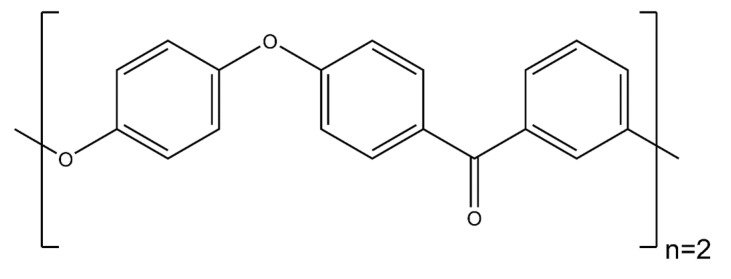
Molecular structure of peek dimer.

The wetting behavior of eight polymer resin systems was simulated in this study:

- **Bismaleimide (BMI)**: This is a two-part system consisting of 4,4′-bismaleimidodiphenylmethane (BMPM) and O, O’-diallyl bisphenol A (DABPA), shown in Figure 1. The stochiometric ratio of the two monomers is 1:1, after Huntsman Matrimid 5292.
- **Benzoxazine**: The Bisphenol-A Benzoxazine monomer is shown in Figure 2. This system is modeled after Huntsman Araldite MT 35610.
- **Difunctional epoxy**: This is a two-part system consisting of diglycidyl ether bisphenol F (DGEBF) and diethyltoluenediamine (DETDA) monomers, as shown in Figure 3. The stochiometric molar ratio of resin to hardener is 2:1. This system is modeled after EPON 862/EPIKURE W.
- **Tetrafunctional epoxy**: This is a two-part system consisting of tetraglycidyl methylene dianiline (TGMDA) and diaminodiphenyl sulfone (DDS) monomers, as shown in Figure 4. The stochiometric ratio of resin to hardener is 1:1. This system is modeled after Solvay CYCOM 977-3.
- **Fluorinated cyanate ester**: The hexafluorobisphenol A cyanate ester monomer is shown in Figure 5. This system is modeled after AroCy F-10.
- **Non-Fluorinated cyanate ester**: The phenol novolac cyanate ester monomer is shown in Figure 6. This system is modeled after Primaset PT 30.
- **PEEK monomer**: The polyether ether ketone monomer is shown in Figure 7.
- **PEEK dimer**: The polyether ether ketone dimer is shown in Figure 8.

The selection of these monomers involves several contrasts that provide insight into the wetting behavior of polymer resins with CNT surfaces. The list of monomers includes a thermoplastic (PEEK) and six thermosets. For the PEEK thermoplastic, the monomer and dimer versions were modeled. The purpose of this comparison is to understand the role of molecular weight in wetting. The two cyanate ester systems were chosen to determine the role of fluorine on the wetting behavior. The two epoxy systems were chosen to establish the role of the epoxide group density on the wetting. The inclusion of one-part thermosets (cyanate esters, benzoxazine) and two-part thermosets (epoxies and BMI) provide insight into the effect of monomer mixtures on the wetting behavior. Finally, it is important to note that all of these resins represent aerospace-grade systems that can be used for a wide range of CNT-based composite applications.

Two carbon-based surfaces were simulated in this study:

- Aromatic surface with pure sp^2^ carbon bonding;
- Aliphatic carbon surface with sp^3^ carbon bonding near the surface and hydrogen terminations at the surface.

A wide range of amorphous carbon structures exist, which contain various levels of three principal carbon forms: sp^2^ carbon (graphite), sp^3^ carbon (diamond), and hydrocarbons [23]. As it is not practical to conduct MD simulations on a large number of different amorphous carbon surfaces, the two simulated carbon surfaces were chosen to provide bounds to understand the wetting response of the resins for both aromatic surfaces and amorphous carbon surfaces. That is, the relative wetting responses of the resins on the aromatic and aliphatic carbon surfaces provides insight into the expected relative wetting responses of the resins on amorphous carbon. It is important to note that the diamond structure of carbon is a bulk structure, and the surface carbon atoms must be bonded to terminal groups to maintain their valency. Hydrogen atoms have been chosen as the terminal groups in this study because hydrocarbons are another principal form of amorphous carbon.

### 2.2. Molecular Modeling

The Large scale Atomic/Molecular Massively Parallel Simulator (LAMMPS) software package was used to perform the MD simulations in this study. The Interface Force Field (IFF) developed by Heinz et al. [24] was utilized. IFF has been parameterized by a surface model database to cover critical surface chemistry, which makes it well-suited for the purpose described herein. Heinz et al. [25] showed that IFF is capable of assigning partial charges on molecules accurately, which is particularly important when simulating material interfaces. IFF has been proven to predict the interfacial properties between polymer matrix and carbon-based reinforcement materials efficiently [8,10]. 

#### 2.2.1. Carbon Surfaces

**Figure 9:**
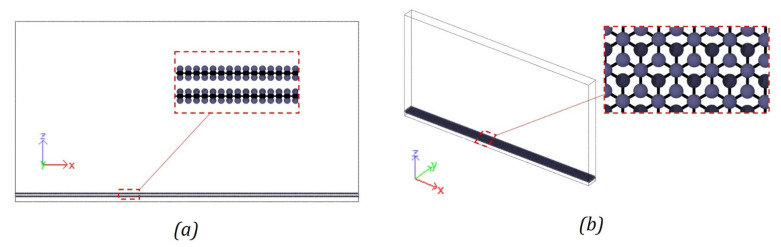
Aromatic carbon surface model with virtual pi electrons (**a**) aromatic carbon surface model with an enlarged view showing Table 1. (**b**) 3D view of the aromatic carbon surface model with an enlarged view in the XY plane.

Previous studies have shown that pseudo-2D simulations for wetting are efficient and capable of generating accurate results [14,15,21]. Surblys et al. [14] predicted the wetting properties of a water–alcohol mixture on a platinum solid surface and demonstrated that a pseudo-2D simulation is optimal to identify the contact angle values. In this study, a similar approach was used to perform contact angle simulations for monomers on flat carbon surfaces. For the aromatic surface, two layers of graphene were modeled in a simulation box of size of ~400 Å × 21 Å × 210 Å (Figure 9). The two layers of graphene were modelled to represent large radii double-walled CNT. The simulation box was periodic in the *x*- and *y*-directions. The positions of the carbon atoms on the graphene planes were obtained by generating an array of atoms in a graphene lattice structure in LAMMPS. A lattice constant of 2.46 Å was used with the primitive lattice vectors given by Gray et al. [26]. Virtual pi electrons were modeled using dummy atoms to capture the effect of the pi electrons on aromatic carbon [8,27]. A detailed discussion about these virtual pi electrons can be found elsewhere [28]. The aromatic carbon surface model consisted of 19,400 atoms including the dummy atoms. 

**Figure 10:**
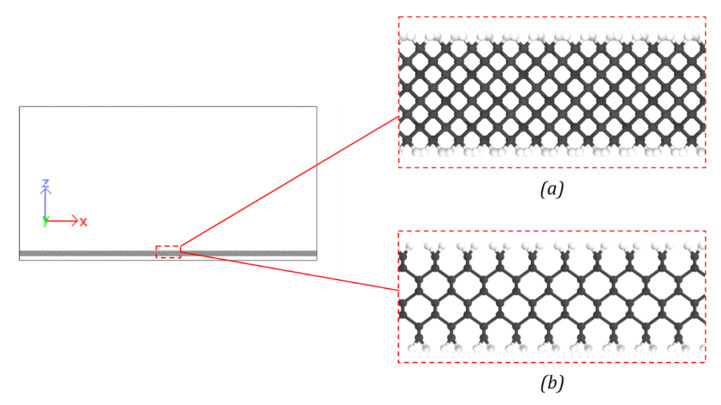
Aliphatic surface model with valency-terminated hydrogen atoms (**a**) enlarged view of the aliphatic carbon surface with valency terminated hydrogen atoms in XZ plane (**b**) enlarged the view of aliphatic carbon surface with valency terminated hydrogen atoms in the XY plane.

A diamond lattice structure was used to create the aliphatic carbon surface model (Figure 10). The thickness of the model was two diamond crystal unit cells. The atomic positions for the diamond lattice structure were obtained with LAMMPS. Hydrogen atoms were added at the top and bottom surfaces of the diamond to balance the valency of the carbon atoms. An energy minimization was performed to relax hydrogen atoms into their equilibrium positions. The final model of the aliphatic surface consisted of 16,416 atoms with a simulation box size of ~406 Å × 20 Å × 210 Å. 

#### 2.2.2. Wetting Simulations

**Figure 11:**
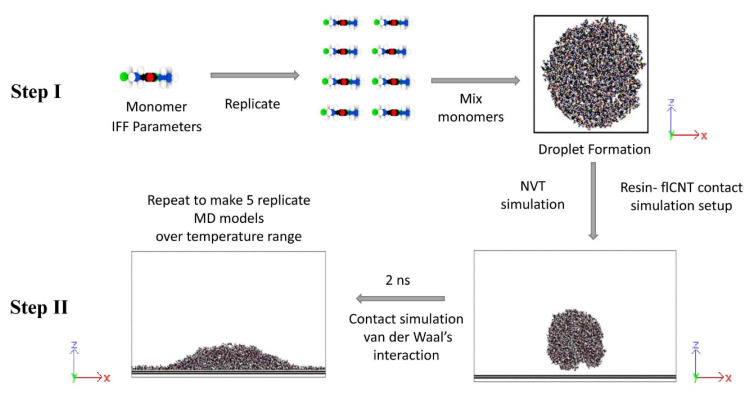
MD workflow for wetting simulations.

The MD wetting simulations consisted of two steps: monomer droplet formation and monomer–surface contact simulation. The modeling steps are schematically represented in Figure 11. The MD framework was verified to determine the simulation parameters by simulating wetting for polystyrene oligomers on an aromatic carbon surface. This is discussed in the Supplementary Materials (SI). The wetting simulations were performed over a range of elevated temperatures that included the typical processing temperatures of the simulated resins. Five replicate models of each monomer system were constructed at each temperature to establish the statistical variation in the predictions.

For the first step (Step I, Figure 11), the resin monomers were constructed, replicated, and placed in an MD simulation box of 20 Å length in the y-direction to form a droplet. Table 1 shows the number of atoms in the monomer droplet models for each resin system. The simulation box size in the x- and z-directions was modified based on the resin system being modelled. The simulation box boundaries were non-periodic and reflective in the x- and z-directions, and periodic in the y-direction. The droplet formation simulations were performed in the NVT ensemble at various simulation temperatures in the range 87–207 °C for 1 ns with a timestep size of 1 fs. 

In the second step of the simulation process (Step II, Figure 11), the resin droplet behavior in the vicinity of the surface was simulated. The resin droplets were placed in the MD simulation boxes containing the aromatic and aliphatic surfaces. The simulation box boundaries were periodic in the x-, y-, and z-directions for this step. The initial distance between the surface and the droplets was 7 Å to allow for non-bonded interactions. Coulombic pairwise interactions and long-range van der Waals interactions were included in these simulations, as given by the Lennard–Jones potential [29]

(1) $$E_{ij}=\underset{i,j}{\sum }\epsilon_{ij}\left[\;2{\left(\;\frac{\sigma_{ij}}{r_{ij}}\;\right)}^{9}\;-3{\left(\;\frac{\sigma_{ij}}{r_{ij}}\;\right)}^{6}\;\right]\;\;\;\;\;\;\;\;\;r\;<\;r_{c}$$

where *r_ij_* is the distance between atoms *i* and *j*, *σ_ij_* is the equilibrium distance between atoms *i* and *j*, ϵ*_ij_* is the well depth parameter which is calculated as the geometric mean of individual atom parameters for atoms *i* and *j*, and *r_c_* is the specified cutoff distance. Columbic interactions were calculated using [30]

(2) $$E\;=\;\frac{Cq_{i}q_{j}}{\epsilon_{0}r_{ij}}\;\;\;\;\;\;\;\;\;\;r\;<\;r_{c}$$

where *r_ij_* is the distance between atoms *i* and *j*; *C* is the energy unit conversion constant; *q_i_* and *q_j_* represents the atomic charges of atoms *i* and *j*, respectively; $\epsilon_{0}$ is the dielectric constant; and *r_c_* is the specified cutoff distance.

**Figure 12:**
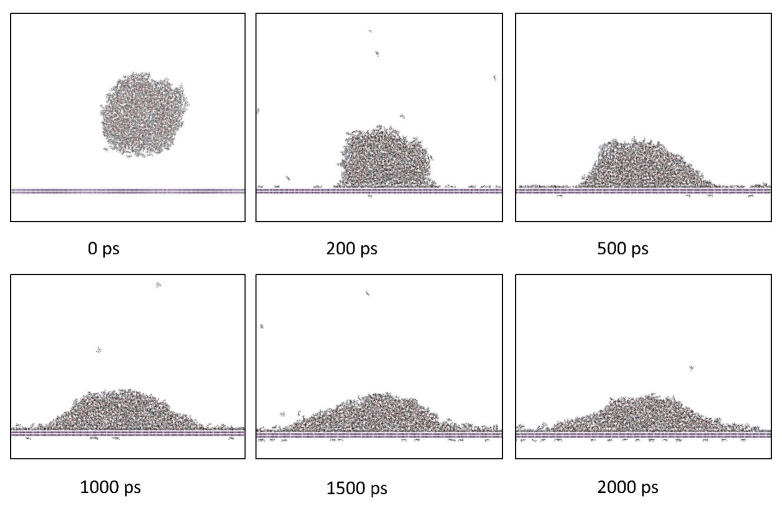
Snapshots of a contact simulation for a representative difunctional epoxy system on an aromatic surface at 147 °C.

These simulations were performed for 2 ns and are henceforth referred as the ‘contact simulations.’ The contact simulations were performed for a simulation time of 2 ns in the NVT ensemble at a simulation temperature selected from the temperature range of 87–207 °C with a time step size of 1 fs. Figure 12 shows simulation snapshots of a representative contact simulation for the difunctional epoxy system on the aromatic surface at 147 °C. The spread of the monomer droplet on the surface can be easily observed over the 2 ns simulation time. 

#### 2.2.3. Evaluation of Contact Angle Value

**Figure 13:**
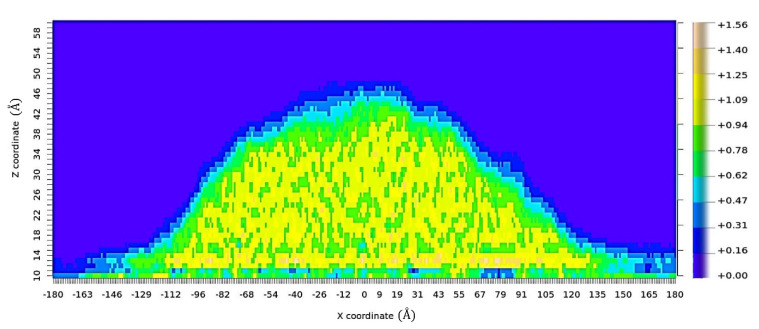
Density map showing density values for the 1 Å × 1 Å grid in g/cc for a difunctional epoxy system on the aromatic surface at 147 °C.

**Figure 14:**
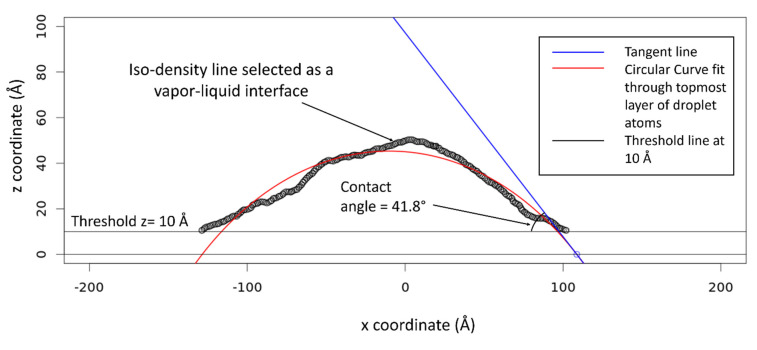
Determination of the contact angle value by fitting an equation of a circle through the topmost layer of monomer droplet atoms.

The contact angle was predicted for each resin system, surface type, and temperature. The method used here to determine the contact angle value is similar to the method developed by Sumith et al. [31]. An array of two-dimensional 1 Å × 1 Å windows were created in the x–z plane of the simulation box, and the average mass density of each window was determined over the final 500 ps of the contact simulations. Using a PYTHON script, this raw density data was processed and rewritten in the form of an array that is readable by RStudio. The resulting mass density map was used to identify the top layer of the monomer droplet. RStudio was used to create a contour-line map from the discrete density points by calculating iso-density lines through the density map. The largest contour line was selected to represent the liquid–vapor interface. Figure 13 shows the density map for the difunctional epoxy system on an aromatic surface at 147 °C at the last timestep of the contact simulation. On the solid surface, low-density monomer vapor molecules were observed near the point of contact as shown in Figure 13. To accurately measure the contact angle value, these mass density values were eliminated by assigning a threshold value in terms of the z-coordinate. This step effectively filtered the density map data below the threshold line at z = 10 Å as shown in Figure 14. The liquid–vapor interface was subjected to a circular fit (red curve, Figure 14). The slope of the tangent (blue line, Figure 14) at the point of intersection between the circular arc and the surface was determined and the corresponding contact angle was calculated. To eliminate the effect of statistical variations in droplet shape the average of the five contact angle values with standard error are presented. Calculated contact angle values are provided in the SI. 

#### 2.2.4. Calculation of Interaction Energy

The interaction energies (IE) between the droplet and the surface were determined in LAMMPS using the “compute group/group” command to gain physical insight on the contact angle values for the aromatic and aliphatic surfaces. In LAMMPS, IE is calculated by

(3) $$IE_{AB}=E\left(A,B\right)-\left(E_{A}+E_{B}\right)$$

where $IE_{AB}$ is the interaction energy between atoms in group A and group B; $E_{A}$ is the total energy of atoms in group A; $E_{B}$ is the total energy of atoms in group B; and E(A,B) is the total energy of entire system including atoms in groups A and B. 

To calculate the IE between the monomers and surface, monomer droplet atoms and surface atoms were assigned to two different groups in LAMMPS. Calculated IE values were averaged over the final 200 ps of the contact simulation. In addition, the atoms of each functional group present in the monomer were assigned to a respective subgroup. IE values between these subgroups and the surface were calculated using Equation (3), where A was assigned as the group of surface atoms and B was assigned as the atoms in a specific functional group in the system. The calculated IE values represent the individual contributions of the surface interaction of specific monomer functional groups towards the total IE values. Higher negative magnitudes of IE indicate higher levels of interaction between the monomers/group and carbon surface.

## 3. Results and Discussions

**Figure 15:**
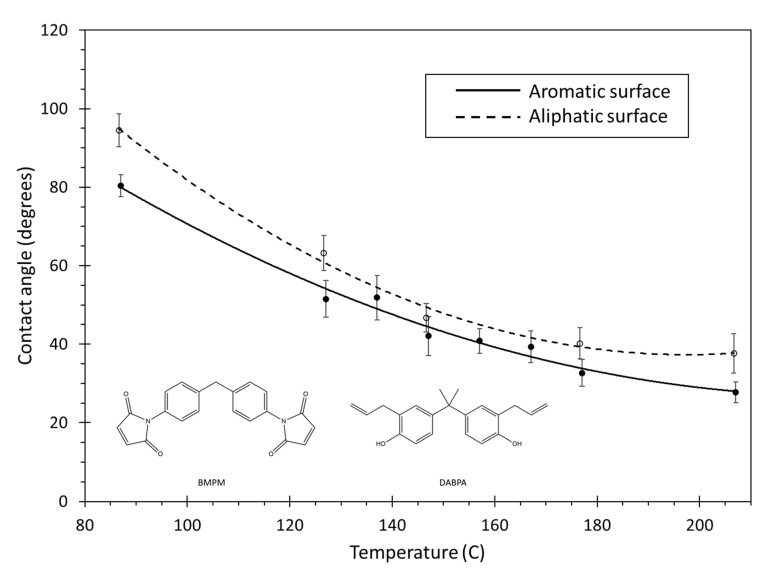
Plot of contact angle vs. temperature for BMI. The lines are curve fits to the data, and the error bars represent the standard error of replicate predictions.

Figure 15 shows the contact angle of the BMI system for both surfaces and the entire range of simulated temperatures. The contact angle generally decreases as the temperature increases, and the contact angle is higher for the aliphatic surface for the entire temperature range. As amorphous carbon is a combination of aromatic and aliphatic carbon groups, the wettability of BMI with amorphous carbon is likely somewhere in-between the aromatic and aliphatic curves. 

It is helpful to interpret the predicted wetting behavior as a competition between adhesion and cohesion. That is, the monomers are attracted to both the carbon surface and to each other. It is the relative strength of these two interactions that dictates how the resin wets onto the carbon surface. Specifically, if the monomer’s interaction with the surface (adhesion) is in some way diminished, then monomer–monomer cohesion becomes dominant and will cause the droplet to form a tighter sphere, increasing the contact angle. If the adhesion is in some way enhanced, then the monomer–monomer cohesion plays a more minor role and the droplet will no longer hold together in a tight sphere, thus decreasing the contact angle.

The decrease in contact angle with increasing temperature shown in Figure 15 can be explained in terms of the monomer flexibility and mobility. Higher temperatures increase monomer flexibility, which allows for access to more conformations that can potentially increase the adhesion of the monomer with the carbon surfaces. Furthermore, higher temperatures increase the thermal motion of the monomers, thus increasing monomer mobility and enabling droplet shape changes. Under laboratory conditions, this effect manifests itself in lower observed resin viscosities at higher temperatures. 

**Figure 16:**
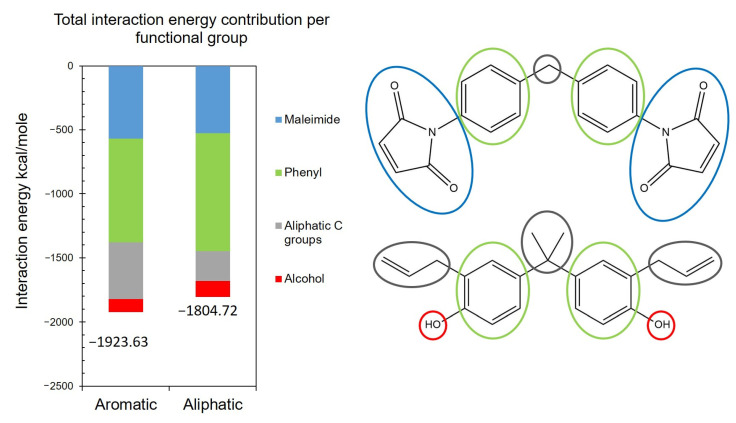
Interaction energy contribution by functional group in the BMI system for both aromatic and aliphatic surfaces.

Figure 16 shows the total IE between the BMI monomers and both carbon surfaces, and the contribution towards the IE by functional group at 207 °C. The data in the figure indicate that the average IE for the five replicate models between the BMI monomers and the aromatic surface is 118.91 kcal/mole higher than that for the aliphatic surface. Close examination of Figure 16 shows that this difference in total IE values is mostly due to the higher interaction of the aliphatic C groups with the aromatic surface. To provide physical insight into this observation, it was determined that the positively charged H atoms (+0.053 C) on the aliphatic surface repel the H atoms in the aliphatic C groups and H atoms in the phenyl rings in the monomers, thereby creating resistance for the droplet to spread on the surface. Furthermore, the negatively charged dummy atoms on the aromatic surface (−0.1 C) attract the H atoms in the monomers, thus improving the wetting of BMI on the aromatic C surface.

**Figure 17:**
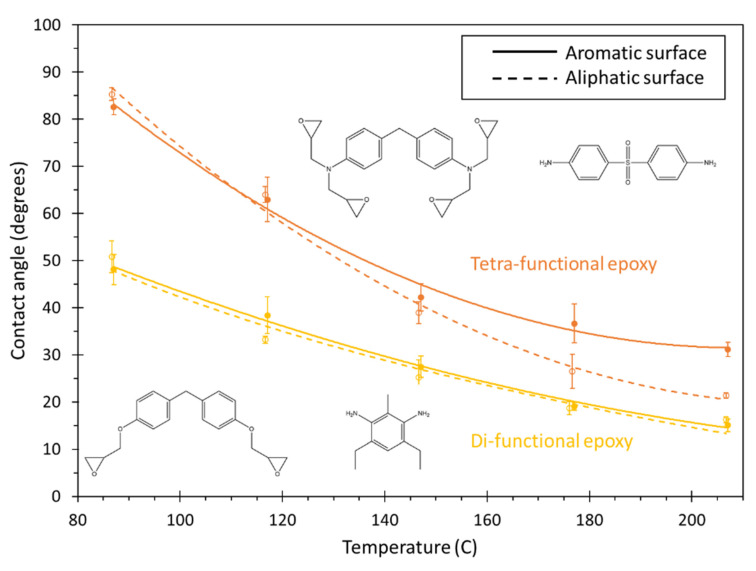
Plot of contact angle vs. temperature for both epoxy systems. The lines are curve fits to the data, and the error bars represent the standard error of replicate predictions.

Figure 17 shows the contact angles for the two epoxy systems for both carbon surfaces as a function of temperature. Similar to the BMI system, both epoxy systems demonstrate an improvement in wetting for increasing temperatures. Whereas the BMI system showed better wetting on aromatic surfaces for the entire temperature range, the di-functional epoxy demonstrates no clear statistical difference in the wetting on the two surfaces, and the tetrafunctional epoxy shows improved wetting on the aliphatic surface for temperatures above about 150 °C. Regardless of the surface, the di-functional epoxy clearly has better wetting than the tetra-functional epoxy for the entire temperature range, which means this trend is expected to hold for amorphous carbon surfaces as well. This is likely because the tetra-functional resin has four epoxide groups per monomer while the di-functional epoxy has only two. This reduces the monomer flexibility of the tetra-functional epoxy system. The resulting steric hinderance reduces the ability of the tetra-functional system to wet onto either surface.

**Figure 18:**
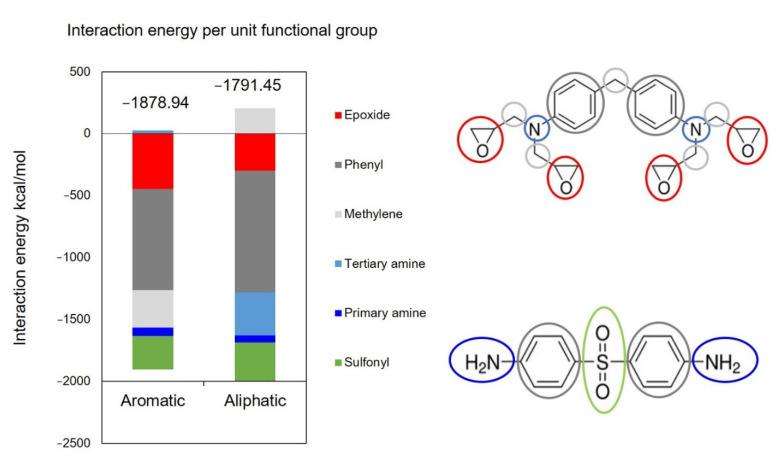
Interaction energy contribution by functional groups in the tetrafunctional epoxy system for the aromatic and aliphatic surfaces.

To investigate improved wettability of tetrafunctional epoxy on the aliphatic surface at elevated temperatures, the IE of each functional group with the surface was determined. Figure 18 shows the IE for the indicated functional groups in the tetrafunctional epoxy system at 207 °C. From Figure 18 it is clear that the interaction of tertiary amines, phenyl groups, and the sulfonyl group is stronger with the aliphatic surface. Even though the epoxide and methylene group interactions are stronger with the aromatic surface, their contributions are not enough for the tetrafunctional epoxy to have stronger interaction with the aromatic surface. The increased overall interaction of the tetrafunctional epoxy with the aliphatic surface at high temperatures is likely due to the increased flexibility and mobility of the monomers at higher temperatures coupled with the strong attraction of the tertiary amine nitrogen atoms (charge of −0.422 C) with the hydrogen atoms on the aliphatic surface (charge of 0.053 C) relative to the virtual electrons on the aromatic surface (charge of −0.1 C). The IE analysis for difunctional epoxy is provided in the SI. 

**Figure 19:**
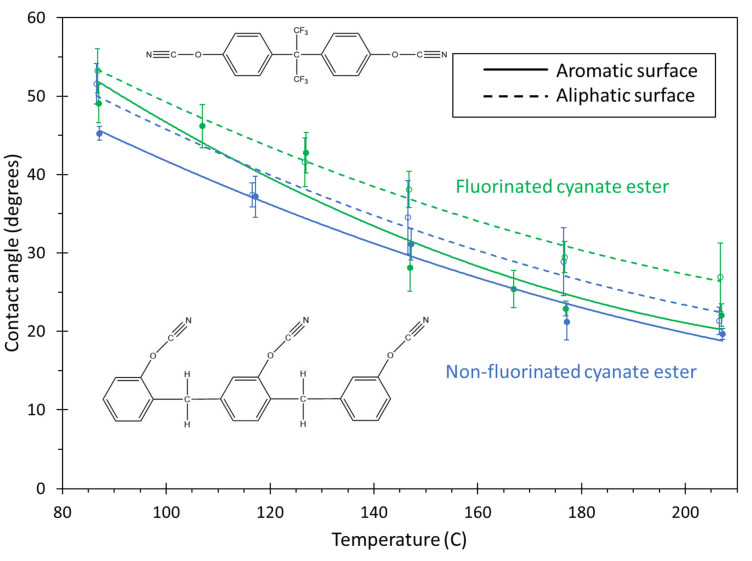
Plot of contact angle vs. temperature for both cyanate ester systems. The lines are curve fits to the data, and the error bars represent the standard error of replicate predictions.

Figure 19 shows the contact angle of the two cyanate ester systems over the full temperature range for both carbon surfaces. As with the BMI and epoxy systems, the contact angle consistently decreases with increases in temperature. For the aromatic surface, the non-fluorinated cyanate ester demonstrates better wettability at temperatures below about 130 °C.

The fluorinated cyanate ester monomers include trifluoromethyl groups that may provide a steric hinderance that restricts mobility, thus providing resistance to the spread of the monomer droplet over the aromatic carbon surface in this temperature range. The contact angles for the two systems on the aromatic surface are nearly the same for temperatures above about 130 °C. For the aliphatic surface, the curve fit line of the non-fluorinated system consistently shows a lower contact angle than that of the fluorinated system, however, examination of the overlap of the error bars indicates that there is no statistical difference in the contact angle of the two systems. Furthermore, considering the overlap of the error bars, there is no consistent difference in the contact angles of either system when comparing the two carbon surfaces. The detailed IE contribution per functional group analysis for both cyanate esters is provided in the SI.

**Figure 20:**
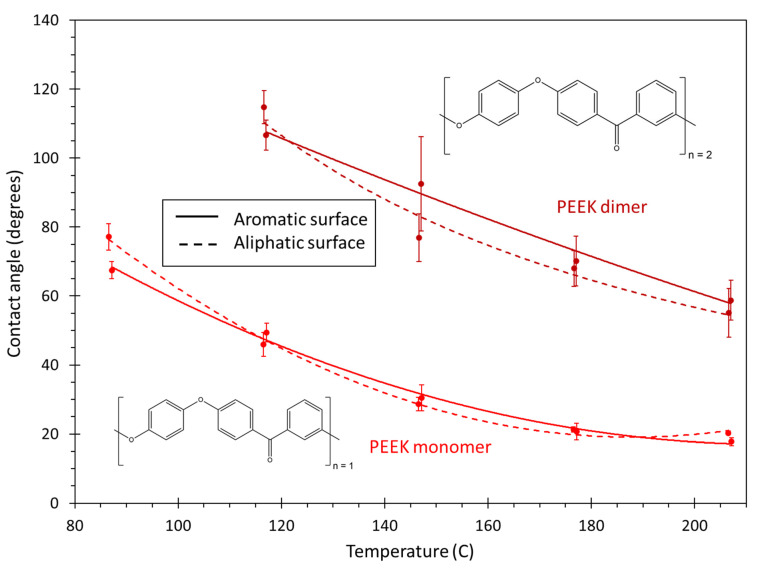
Plot of contact angle vs. temperature for PEEK. The lines are curve fits to the data, and the error bars represent the standard error of replicate predictions.

Figure 20 shows the contact angle of the PEEK monomer and dimer. As with the other polymer systems, PEEK shows a decrease in contact angle with an increase in temperature. For the full temperature range, the PEEK monomer clearly has a lower contact angle than the PEEK dimer. This indicates that the contact angle values are highly chain-length dependent. This is likely a direct cause of the increased steric hindrance associated with the longer chain lengths of the PEEK dimer. Neither the PEEK monomer nor dimer show a significant effect on the wettability with respect to the carbon surface type. Therefore, it is expected that both PEEK systems will demonstrate similar trends with wetting on amorphous carbon surfaces. The IE analysis for both PEEK systems is provided in the SI.

**Figure 21:**
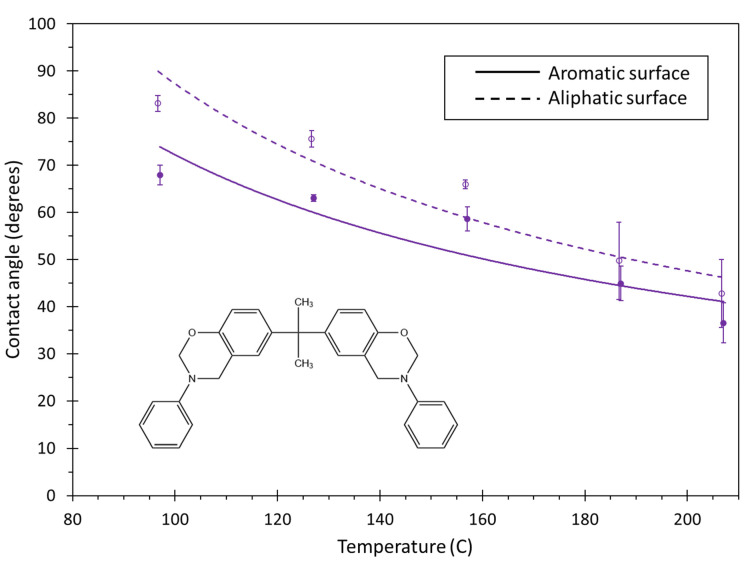
Plot of contact angle vs. temperature for benzoxazine. The lines are curve fits to the data, and the error bars represent the standard error of replicate predictions.

Figure 21 shows the contact angle of the benzoxazine monomers over the full temperature range on both carbon surfaces. As with the other monomers, the contact angle decreases with increasing temperatures. The contact angle values between the two surfaces shows a difference of 13 °C at 127 °C. For higher temperatures, the overlap of the error bars indicates that there is no statistical difference in the contact angle on the two surfaces. IE analysis for benzoxazine is provided in the SI.

**Figure 22:**
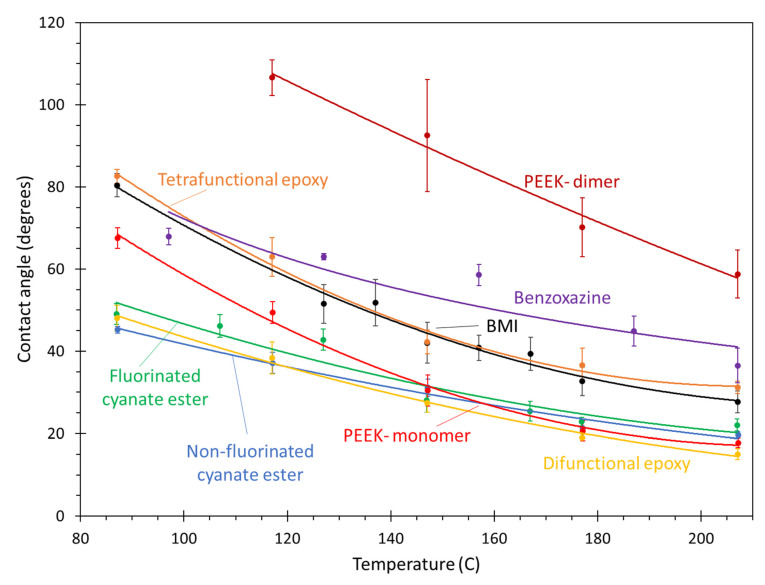
Plot of contact angle vs. temperature of monomers on the aromatic surface. The lines are curve fits to the data, and the error bars represent the standard error of replicate predictions.

Figure 22 shows the contact angle values of the polymers on the aromatic surface as a function of temperature. In general, the PEEK monomer, difunctional epoxy, and both cyanate ester systems show the best overall wettability over the temperature range. The tetrafunctional epoxy, BMI, and benzoxazine systems demonstrate an intermediate wettability, while the PEEK dimer clearly shows lower relative wettability. These trends are caused by a balance between monomer size, flexibility, and interaction with the aromatic surface. That is, larger, less flexible monomers have more steric hindrance and cannot conform easily to the aromatic surface. There is a thermodynamic drive for aromatic groups in the monomers to align themselves with the aromatic carbon surface because of the pi–pi interaction [32,33,34,35] (as simulated using the virtual pi electrons described above), which can increase the overall monomer/surface interaction and thus lower the contact angle. However, the steric hindrance from inflexible regions of the monomers can inhibit this alignment. Specifically, the PEEK monomer, difunctional epoxy, and cyanate ester systems all have relatively small and flexible monomers that contain aromatic groups located in flexible portions of the monomer chain. For the tetrafunctional epoxy, BMI, benzoxazine, and PEEK dimer systems, the monomers are slightly larger and/or less flexible, which prevents them from fully conforming to the aromatic surface and thus lowers their wettability.

Other important information can be determined from the data in Figure 22. Regarding the influence of the trifluoromethyl groups on wetting onto aromatic surfaces, there is a negligible difference in the contact angle between the fluorinated and non-fluorinated cyanate esters relative to the magnitudes of contact angles of the other resins. Additionally, while the PEEK monomer shows excellent wettability, the contact angle of the PEEK dimer is significantly higher than the other resins. This demonstrates that the size of thermoplastic chains has a significant impact on wettability. Therefore, as is already well-known in the composites processing community, lower molecular weights of thermoplastic polymers result in improved processability. In practice, composite processing does not generally occur with PEEK in monomeric form. Therefore, the most significant result of Figure 22 is the excellent wettability of the cyanate esters and difunctional epoxy onto the aromatic surface. 

**Figure 23:**
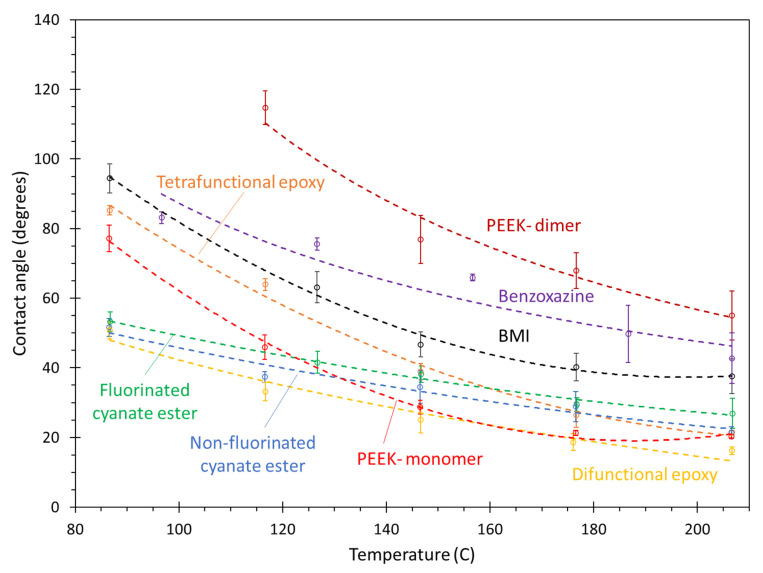
Plot of contact angle vs. temperature of monomers on the aliphatic surface. The lines are curve fits to the data, and the error bars represent the standard error of replicate predictions.

Figure 23 shows the contact angle of the monomers on the aliphatic surface as a function of temperature. Below 150 °C, the PEEK monomer, difunctional epoxy, and cyanate esters clearly show the lowest contact angles, with the tetrafunctional epoxy, BMI, benzoxazine demonstrating intermediate amounts of wetting, and the PEEK dimer with the relatively lowest wetting. This trend continues above 150 °C with the exception of the tetrafunctional epoxy, which demonstrates a significant increase in wetting, on par with the difunctional epoxy, cyanate esters, and PEEK monomer. These overall trends in the monomers likely result from steric hindrance associated with larger and less flexible monomers and the non-bonded attraction with the aliphatic surface and specific chemical groups in the monomer, as described above. 

Other important conclusions can be discerned from Figure 23. Similar to the aromatic surface results, the influence of the trifluoromethyl groups in the cyanate ester systems on contact angle is not significant. Additionally, the influence of thermoplastic chain size on the contact angle is significant given the wettability demonstrated by the PEEK monomer and dimer, as was observed with the aromatic surface results. As PEEK monomers are not generally used for composite processing, the most significant results shown in Figure 23 are the excellent wettability of the difunctional epoxy and cyanate ester systems over the entire temperature range, and the excellent wettability of the tetrafunctional epoxy onto the aliphatic surface at temperatures above 150 °C.

**Figure 24:**
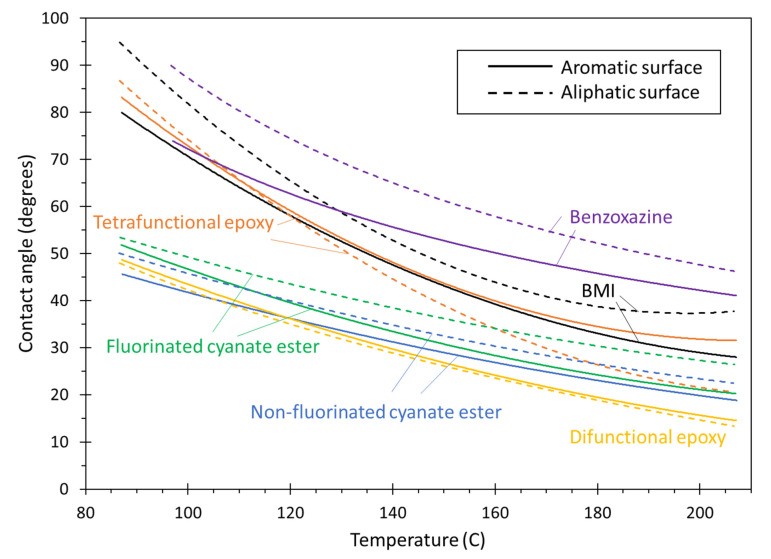
Plot of contact angle vs. temperature of thermosets on both surfaces. The lines are curves fit to the data, and individual averages and error bars have been removed for clarity.

Figure 24 shows the trend lines for all of the thermoset systems on both the aromatic and aliphatic surfaces. Although the comparisons for each individual resin on both surfaces are discussed above, this figure highlights the relative differences in the wetting on the two surfaces with respect to the entire set of thermosetting resins. Specifically, the figure indicates that the different responses on the two surfaces for the tetrafunctional epoxy at higher temperatures, the benzoxazine at lower temperatures, and the BMI over the entire temperature range are far greater than difference in responses for the other thermosetting resins. 

Figure 24 is also helpful for estimating the relative performance of the thermoset systems on amorphous carbon surfaces. If the amorphous carbon surface wetting trendline is assumed to be somewhere between the amorphous and aliphatic trendlines, then it is clear that, at relatively low temperatures, the difunctional epoxy and cyanate ester systems will exhibit the highest levels of wetting on amorphous carbon surfaces. At high temperatures (above 150 °C), the difunctional epoxy exhibits the overall highest wetting on amorphous carbon, with the cyanate esters and tetrafunctional epoxy close behind. The BMI and benzoxazine are expected to show lower levels of wetting on amorphous carbon surfaces at these temperatures.

## 4. Conclusions

An MD framework was developed to predict the contact angle values of polymer resins on CNT surfaces dominated by aromatic carbon, aliphatic carbon, or a mixture thereof (amorphous carbon). The monomer chain length, temperature, and the functional groups in the monomers have a significant impact on the contact angle values on the CNT surface. An overall trend of decreasing contact angle (improvements in wetting) with increasing temperature was observed for all the resins studied. 

Considering the results from this study, the difunctional epoxy demonstrates the best wettability of the thermoset resins considered herein, regardless of the CNT material surface structure. However, for applications that require relatively high temperatures, the difunctional epoxy may be unsuitable due to its lower glass transition temperature relative to the tetrafunctional epoxy [36,37] and the cyanate ester resins become the best option in terms of wettability on any CNT material surface. For higher processing temperatures, the tetrafunctional epoxy is also a good candidate for resin selection, particularly for CNT materials that have amorphous carbon on the surface. The results of this study also indicate that PEEK in its monomer form has excellent wettability on CNT surfaces, but PEEK in the dimer form has wetting capabilities below those of the thermosets studied. As composites are not typically processed with monomeric PEEK, higher molecular weights of PEEK are not recommended for optimal wetting in CNT composite fabrication.

## Figures and Tables

**Table 1:** Number of atoms in droplet models.

Resin	Number of Atoms in Droplet Model
Bismaleimide	15,488
Benzoxazine	15,210
Difunctional epoxy	15,444
Tetrafunctional epoxy	15,210
Fluorinated cyanate ester	15,400
Non-fluorinated cyanate ester	14,960
PEEK monomer	14,960
PEEK dimer	14,700

## Data Availability

The data presented in this study are available on request from the corresponding author. The data are not publicly available because they are currently being used for further studies.

## Supplementary Materials

The following are available online at https://www.mdpi.com/article/10.3390/polym13132162/s1, Figure S1: Snapshots of contact angle simulation for PS oligomers at 2 ns, Figure S2: Snapshot of the contact simulation at 2 ns for a representative EPON 862: DETDA system on the aromatic surface at 147 °C, Figure S3: Interaction energy distribution by functional group in the difunctional epoxy system with the aromatic and aliphatic surfaces, Figure S4: Interaction energy distribution by functional group in the fluorinated cyanate ester system with the aromatic and aliphatic surface, Figure S5: Interaction energy distribution by functional group in the non-fluorinated cyanate ester system with the aromatic and aliphatic surface, Figure S6: Interaction energy distribution by functional group in the peek monomer system with the aromatic and aliphatic surface, Figure S7: Interaction energy distribution by functional group in the peek monomer system with the aromatic and aliphatic surface, Figure S8: Interaction energy distribution by functional group in the benzoxazine system with aromatic and aliphatic surface Table S1: Contact angle values of polymers on the aromatic surface, Table S2: Contact angle values of polymers on the aliphatic surface.
