# Supplementary material for: Wetting Simulations of High-Performance Polymer Resins on Carbon Surfaces as a Function of Temperature Using Molecular Dynamics
Source: Polymers (Basel). 2021 Jun 30;13(13):2162. doi: 10.3390/polym13132162 (PMC8271784; doi:10.3390/polym13132162)
Supplement: Supplementary file 1 [file polymers-13-02162-s001.zip › polymers-1268590-supplementary.pdf]

Supplementary Information for

Wetting Simulation of High-Performance Polymers on Carbon Nanotube Surfaces as a Function of Temperature using Molecular Dynamics

Swapnil S. Bamane, Prashik S. Gaikwad, Matthew S. Radue, S. Gowtham, Gregory M. Odegard
Michigan Technological University, Houghton, MI 49931

1. Verification of MD simulation framework

To develop and test the MD wetting simulation protocols, oligomers of polystyrene (PS) monomer (PS1), dimer (PS2), trimer (PS3), and quadmer (PS4) were simulated at room temperature (300 K). This case study determined simulation details such as the number of atoms required to form a droplet, size effect of the monomer chain on droplet formation, and final shapes after contact with the surface. Figure S1 shows the results for the PS oligomers after the contact simulations. The contact angle value changed as the size of oligomer increased. Oligomers with lower molecular weights show good wettability as compared to the longer chains with higher molecular weight, as observed in experimental studies of wrapping of lower mass faction PS chains around CNTs[1-3].

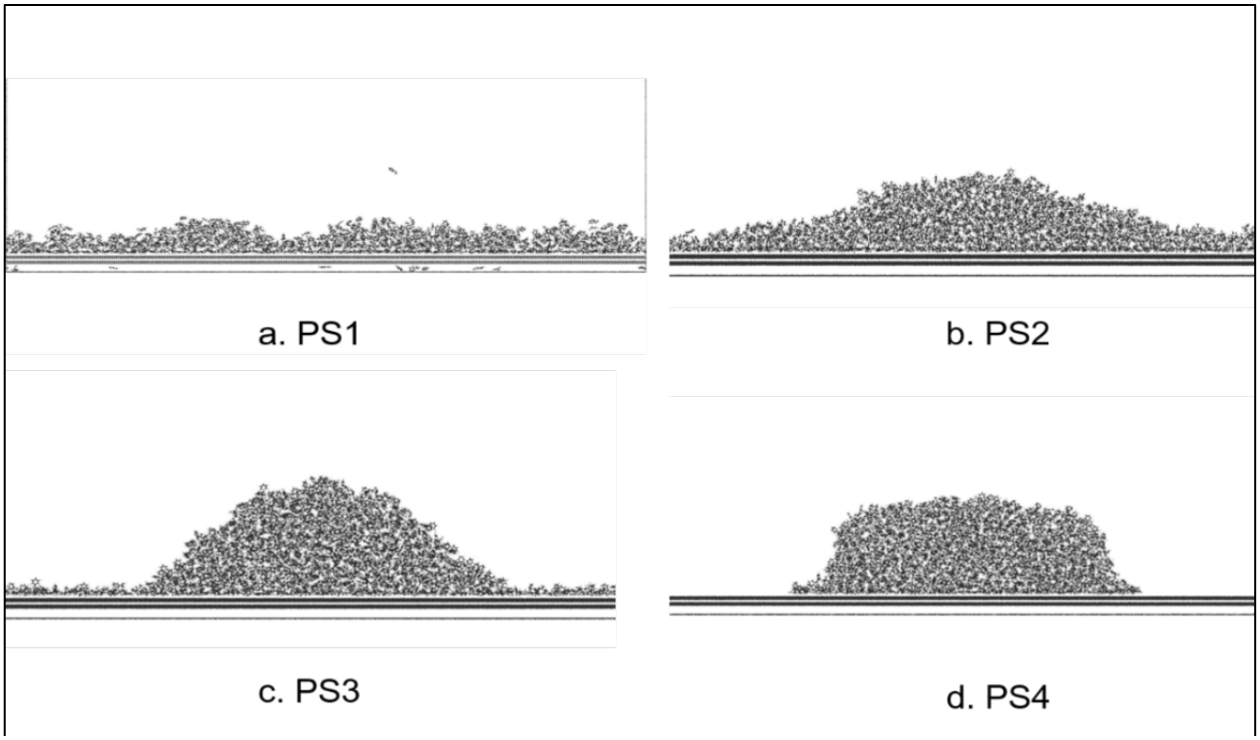

Figure S1: Snapshots of contact angle simulation for PS oligomers at 2ns

2. Contact Angle Simulation Snapshot

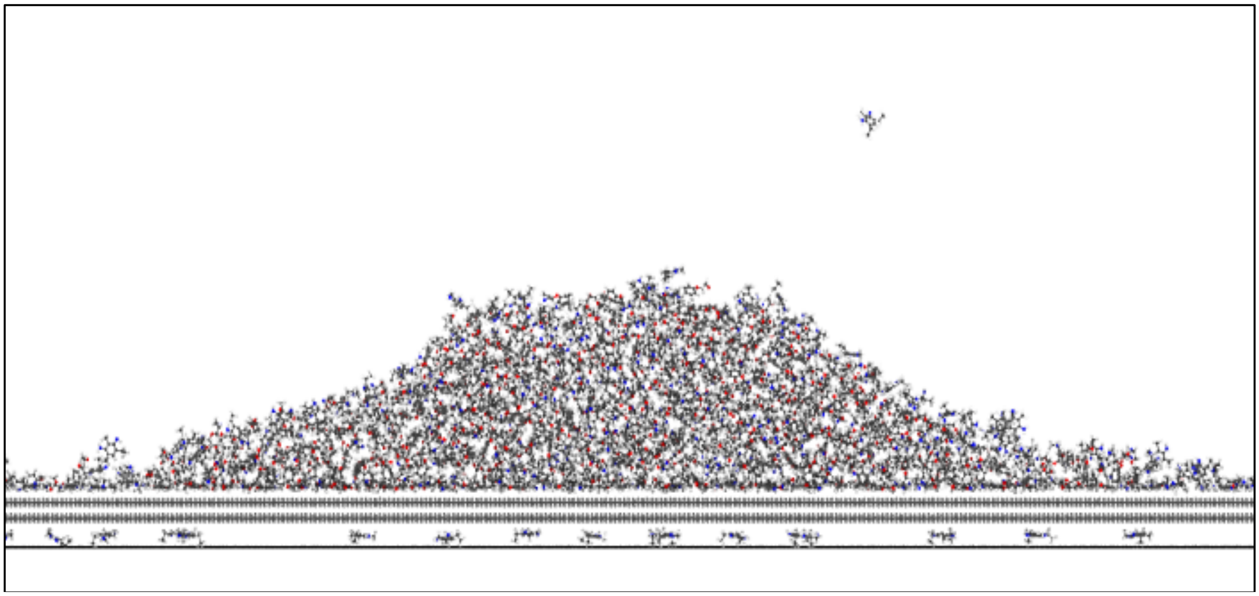

Figure S2: Snapshot of the contact simulation at 2 ns for a representative EPON 862: DETDA system on the aromatic surface at 147 °C

Figure S2 shows a snapshot of a contact simulation performed for the difunctional epoxy system. Low density vapor molecules can be easily seen near aromatic surface. It is also clearly visible that some low-density polymer molecules leave the droplet and float around into the simulation box. These molecules were eliminated from the consideration when calculating the contact angle values. The elimination was performed based on density map generated.

3. Contact angle values

Table S1 shows the contact angle values and the standard deviation for each polymer over the selected temperature range on the aromatic surface.

Table S1: Contact angle values of polymers on the aromatic surface

Polymer	Contact Angle values on sp2 C surface (degree)				
	360 K	390 K	420 K	450 K	480 K
BMI	80.42 ± 2.78	51.52 ± 4.7*	42.06 ± 4.94	32.66 ± 3.46	27.7 ± 2.62
EPOXY (TGMDA:DDS)	82.62 ± 1.7	62.96 ± 4.68	42.24 ± 2.91	36.65 ± 4.11	31.16 ± 1.5
Cyanate Ester (AroCy F-10)	49.06 ± 2.48	42.8 ± 2.59*	28.08 ± 2.96	22.9 ± 0.96	22.06 ± 1.41
Cyante Ester (Primaset PT 30)	45.22 ± 0.87	37.16 ± 2.62	31.14 ± 2.02	21.2 ± 2.34	19.68 ± 0.68
EPOXY (EPON 862:DETDA)	48.8 ± 3.16	38.38 ± 3.88	27.46 ± 2.54	19.04 ± 0.8	15.03 ± 1.3
PEEK-Monomer	53.58 ± 3.23	35.52 ± 0.72	26.08 ± 3.72	20.44 ± 1.37	16.48 ± 0.64
PEEK-Dimer	-	106.63 ± 4.32	92.54 ± 13.64	70.16 ± 7.2	58.78 ± 5.83
	370 K	400 K	430 K	460 K	480 K
Benzoxazine	97.05 ± 2.06	63 ± 0.7	58.56 ± 2.57	44.93 ± 3.66	36.52 ± 4.25

* Contact angle values were calculated at 400 K

Table S2 shows the contact angle values and the standard deviation for each polymer system over the selected temperature range on the aliphatic surface.

Table S2: Contact angle values of polymers on the aliphatic surface

Polymer	Contact Angle values on sp3 surface (degree)				
	360 K	390 K	420 K	450 K	480 K
BMI	94.46 ± 4.2	63.16 ± 4.48*	46.7 ± 3.59	40.2 ± 3.96	37.62 ± 4.98
EPOXY (TGMDA:DDS)	85.28 ± 1.34	63.96 ± 1.69	38.92 ± 2.35	26.46 ± 3.57	21.36 ± 0.64
Cyanate Ester (AroCy F-10)	53.24 ± 2.82	41.58 ± 3.11*	38.12 ± 2.31	29.44 ± 1.99	26.92 ± 4.36
Cyante Ester (Primaset PT 30)	51.58 ± 2.57	37.4 ± 1.54	34.54 ± 4.69	28.86 ± 4.34	21.36 ± 1.75
EPOXY (EPON 862:DETDA)	50.8 ± 2.03	33.18 ± 2.97	25.16 ± 1.96	18.7 ± 1.50	16.23 ± 1.83
PEEK-Monomer	51.48 ± 2.12	37.16 ± 3.29	27.4 ± 3.85	23.2 ± 3.44	17.92 ± 0.92
PEEK-Dimer	-	114.75 ± 4.81	76.9 ± 6.87	67.98 ± 5.15	55.08 ± 7.04
	370 K	400 K	430 K	460 K	480 K
Benzoxazine	83.13 ± 1.71	75.58 ± 1.77	65.95 ± 0.95	49.7 ± 8.22	42.75 ± 7.23

* Contact angle values were calculated at 400 K

4. Interaction Energy Analysis

- **Difunctional epoxy**

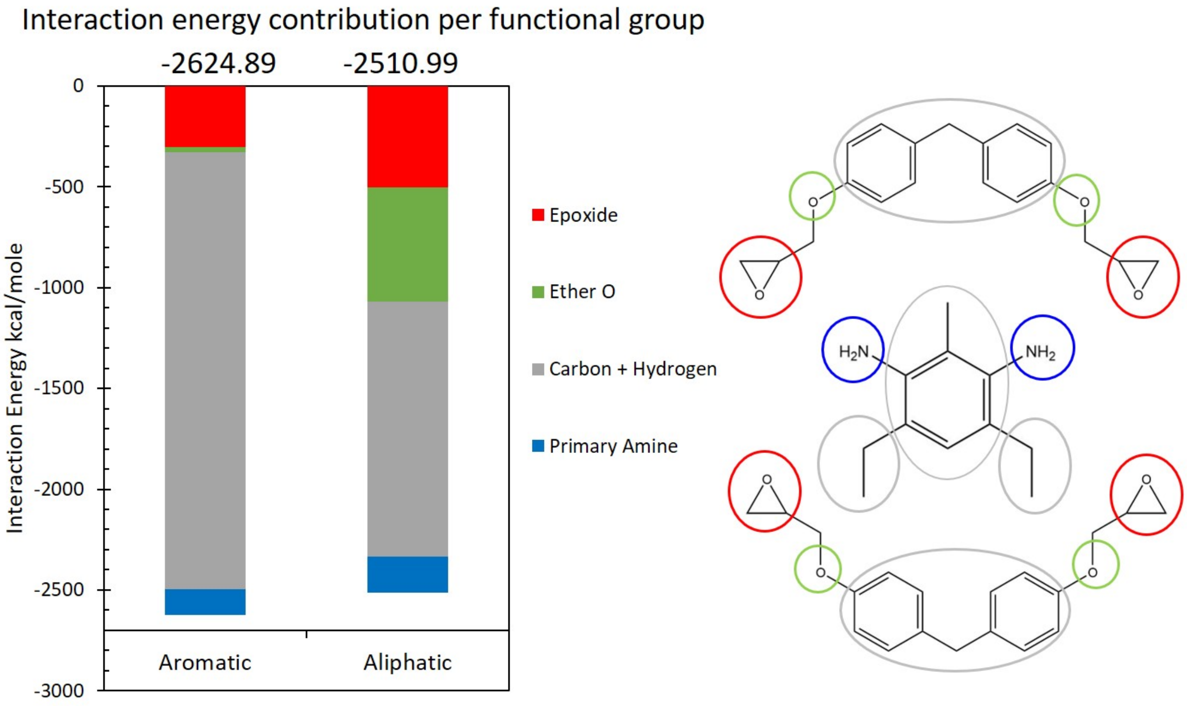

Figure S3: Interaction energy distribution by functional group in the difunctional epoxy system with the aromatic and aliphatic surfaces

From Figure S3, it is clear that there is a significant interaction between the ether O on the aliphatic surface, which is somewhat balanced with the greater interaction of the hydrocarbons in the monomer onto the aromatic surface. As a result, the contact angle values of the difunctional epoxy are within the statistical deviation, and thus the difunctional epoxy does not show a particular surface preference.

- **Fluorinated cyanate ester**

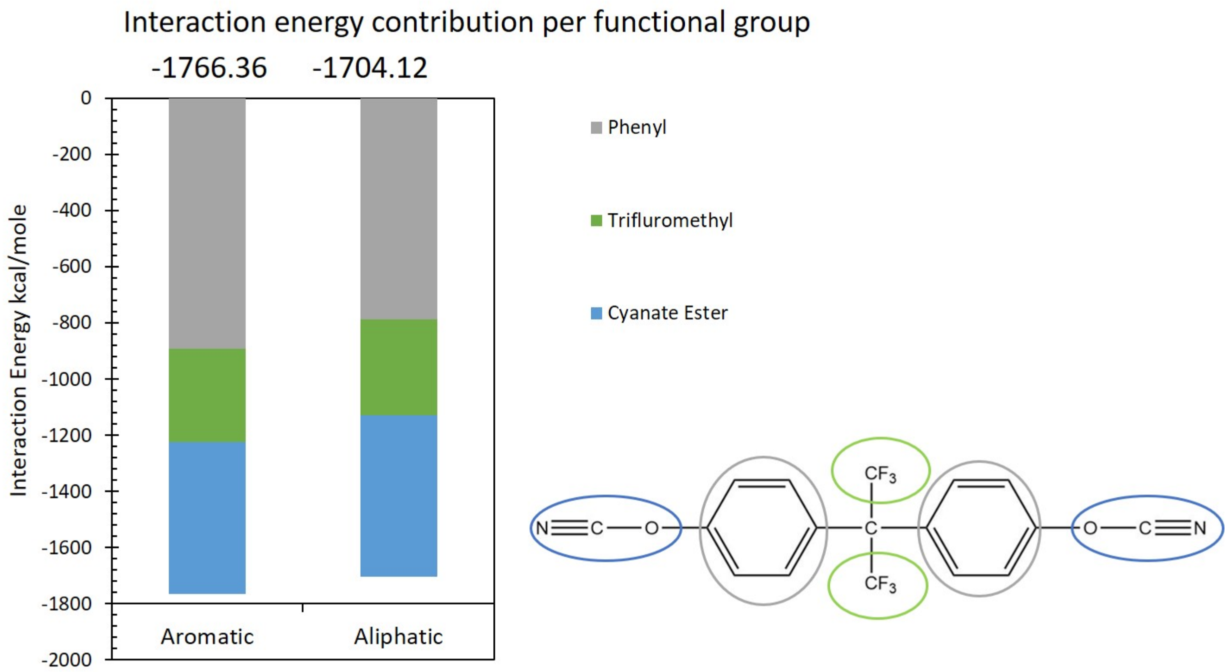

Figure S4: Interaction energy distribution by functional group in the fluorinated cyanate ester system with the aromatic and aliphatic surface

From Figure S*4*, it is clear that there is no significant difference in IE contribution by trifluoromethyl groups and cyanate ester groups on aromatic and aliphatic carbon surfaces. There is a decrease in the IE contributed by phenyl groups by 105 kcal/mole on the aliphatic carbon surface. This decrease in IE contribution explains the better wettability onto the aromatic surface for fluorinated cyanate ester.

- **Non-fluorinated cyanate ester**

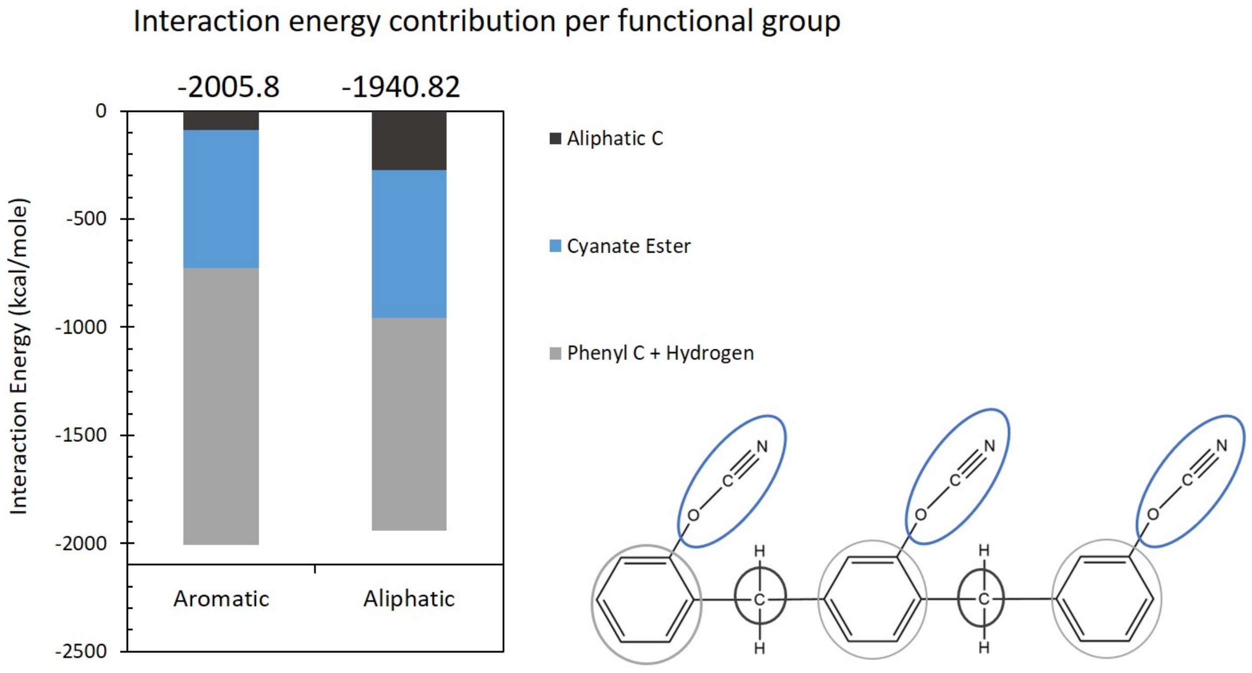

Figure S5: Interaction energy distribution by functional group in the non-fluorinated cyanate ester system with the aromatic and aliphatic surface

Figure S5 indicates that the IE energy contribution between surfaces and non-fluorinated cyanate ester is highly dependent on phenyl groups. It is clear from Figure S5 that the IE contribution of phenyl rings is higher on the aromatic surface. This is likely due to the pi-pi stacking phenomenon of phenyl rings on the aromatic carbon structures, hence the lower contact angle values on aromatic surface than aliphatic surface.

- **PEEK**

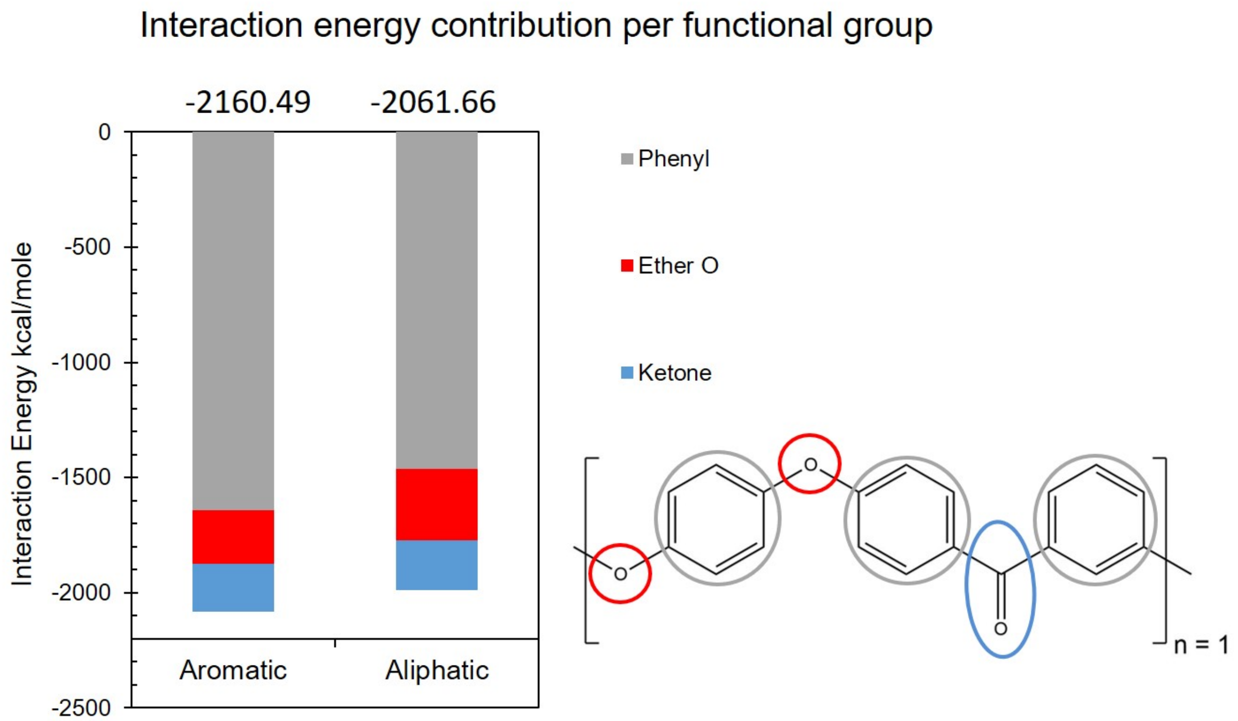

Figure S6: Interaction energy distribution by functional group in the peek monomer system with the aromatic and aliphatic surface

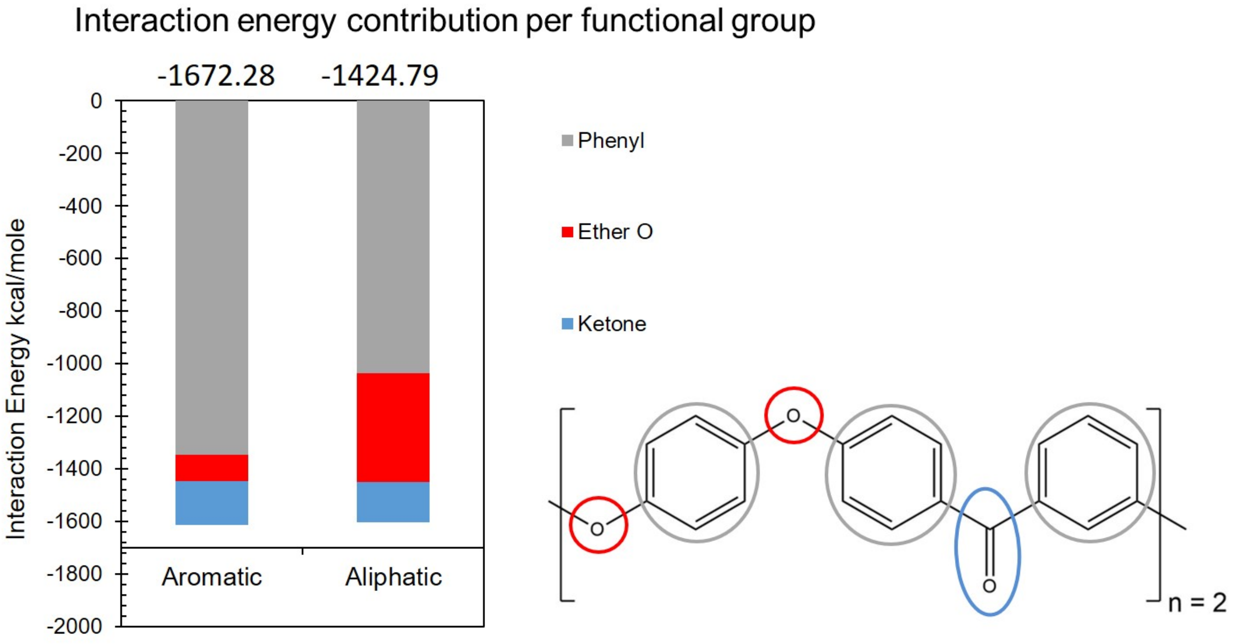

Figure S7: Interaction energy distribution by functional group in the peek monomer system with the aromatic and aliphatic surface

From Figure S6 and Figure S7, we can clearly see that similar to cyanate esters, the IE energy of PEEK is highly dependent on the IE between the surface and phenyl rings in the system. Both monomer and dimer PEEK systems show a slight increase in IE contribution due to oxygen atoms (i.e., ether group present in the chain). On the other hand, Figure S6 shows that there is a decrease in the IE contribution by phenyl rings on the aliphatic surface by 178.8 kcal/mole. Figure S7 shows similar observations with a decrease in IE contributed by phenyl rings on the aliphatic surface by 307.7 kcal/mole. This demonstrates that there is preferential interaction between phenyl groups with the aromatic surface in both monomer and dimer systems of PEEK.

- **Benzoxazine**

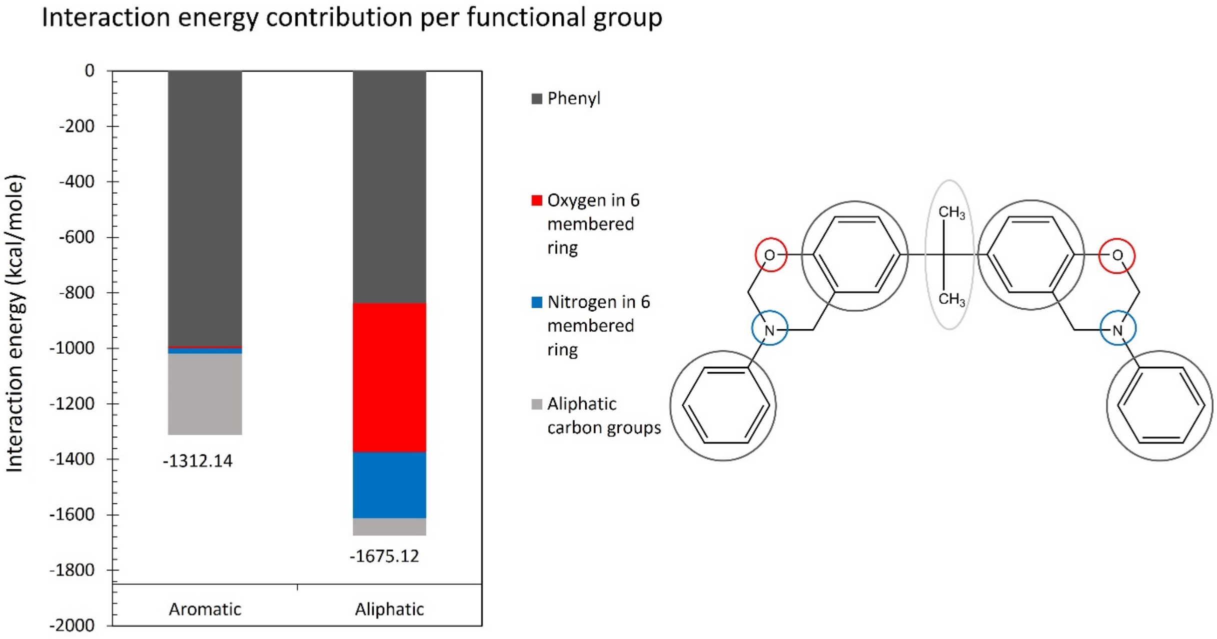

Figure S8: Interaction energy distribution by functional group in the benzoxazine system with aromatic and aliphatic surface

Figure S8 shows the IE comparison between the benzoxazine resin droplets with aromatic and aliphatic surface. It is clear that the IE between nitrogen and oxygen atoms in 6-membered ring is increased significantly with aliphatic surface. This is driven by the coulombic attractions due to charge polarities present in positively charged hydrogen atoms in the aliphatic surface model and negatively charged nitrogen and oxygen atoms in the resin droplet model. However, it is important to note that, even if IE value of droplet with surface is higher with aliphatic carbon surface, contact angle values are lower with aromatic surface. This is observed due to the flexibility of the monomer and conformations of the monomer with the surface. Bulky phenyl rings obstruct the alignment of favored oxygen and nitrogen atoms with aliphatic surface and resulting in higher contact angle values instead. On the other hand, phenyl rings align with aromatic carbon surface and assists droplet to spread out on the surface.

References

1. Grossiord, N.; Miltner, H. E.; Loos, J.; Meuldijk, J.; Van Mele, B.; Koning, C. E., On the Crucial Role of Wetting in the Preparation of Conductive Polystyrene−Carbon Nanotube Composites. *Chemistry of Materials* **2007,** *19* (15), 3787-3792.

2. Yang, M.; Koutsos, V.; Zaiser, M., Interactions between Polymers and Carbon Nanotubes: A Molecular Dynamics Study. *The Journal of Physical Chemistry B* **2005,** *109* (20), 10009-10014.
3. Carlier, V.; Sclavons, M.; Jonas, A. M.; Jérôme, R.; Legras, R., Probing Thermoplastic Matrix−Carbon Fiber Interphases. 1. Preferential Segregation of Low Molar Mass Chains to the Interface. *Macromolecules* **2001,** *34* (11), 3725-3729.
